# Selumetinib - a potential small molecule inhibitor for osteoarthritis treatment

**DOI:** 10.3389/fphar.2022.938133

**Published:** 2022-09-27

**Authors:** Xiaohang Zheng, Jianxin Qiu, Wenjun Pan, Yuhang Gong, Weikang Zhang, Ting Jiang, Lihua Chen, Weifu Chen, Zhenghua Hong

**Affiliations:** ^1^ Orthopedic Department, Taizhou Hospital Affiliated to Wenzhou Medical University, Linhai, China; ^2^ Enze Medical Research Center, Taizhou Hospital Affiliated to Wenzhou Medical University, Linhai, China

**Keywords:** osteoarthritis, selumetinib, necroptosis, osteoclasts, small molecule inhibitor

## Abstract

**Objectives:** Osteoarthritis (OA) is a common disease that mainly manifests as inflammation and destruction of cartilage and subchondral bone. Recently, necroptosis has been reported to play an important role in the development of OA. Selumetinib displays a contrasting expression pattern to necroptosis-related proteins. The present study aimed to investigate the potential therapeutic effects of selumetinib in OA process.

**Methods:** *In vitro* experiments, interleukin-1β (IL-1β) was used to induce necroptosis of chondrocytes. We used high-density cell culture, Western Blot and PT-PCR to observe the effect of different concentrations of selumetinib on the extracellular matrix of cartilage. Afterwards, we visualized the effect of selumetinib on osteoclast formation by TRAP staining and F-actin rings. *In vivo* experiment, we induced experimental osteoarthritis in mice by surgically destabilizing the medial meniscus (DMM) while administering different concentrations of selumetinib intraperitoneally.

**Results:** Selumetinib promoted cartilage matrix synthesis and inhibited matrix decomposition. We found that selumetinib exerted a protective function by inhibiting the activation of RIP1/RIP3/MLKL signaling pathways in chondrocytes. Selumetinib also inhibited the activation of RANKL-induced NF-κB and MAPK signaling pathways in BMMs, thereby interfering with the expression of osteoclast marker genes. In the DMM-induced OA model, a postsurgical injection of selumetinib inhibited cartilage destruction and lessened the formation of TRAP-positive osteoclasts in subchondral bone.

**Conclusion:** Selumetinib can protect chondrocytes by regulating necroptosis to prevent the progression of OA and reduce osteoclast formation. In summary, our findings suggest that selumetinib has potential as a therapeutic agent for OA.

## Introduction

Osteoarthritis (OA), a widespread and disabling disease, often causes pain and limited mobility for patients and thus places a significant burden on the affected individuals and the government health care system (Safiri et al., 2020). Many factors such as trauma, obesity, inflammation, and age have been confirmed to be play a role in the progression of OA (Aigner and Richter, 2012; Turkiewicz et al., 2017). The disease process of OA accelerates with early osteoclast activity and bone remodeling, and then progressively develops into bone formation reduction, cartilage destruction, osteophyte formation, and structural changes in ligaments and joints of surrounding muscles. Multiple inflammatory factors are involved in the disruption of metabolic homeostasis of chondrocytes (Aigner and Richter, 2012; Turkiewicz et al., 2017) affect the synthesis of extracellular matrix (ECM) and proinflammatory cytokines (Beier et al., 2014; Makki and Haqqi, 2017; Na et al., 2020), such as interleukin-1β (IL-1β) and tumor necrosis factor-α (TNF-α). Therefore, the inhibition of the IL-1β-induced inflammatory response may be an effective approach to prevent the progression of OA.

Necroptosis is characterized by the activation of factors associated with damage, which induces a strong inflammatory activation of the extracellular environment. As a pathophysiological form of cell death, necroptosis is frequently involved in the development of various diseases. (Cheng et al., 2021; Yang et al., 2021). Receptor-interacting protein kinase 1 (RIP1) binds to receptor-interacting protein kinase 3 (RIP3) to form a necrosome complex in the early stages of necroptosis and can recruit the phosphorylated effector protein mixed-lineage kinase domain-like protein (MLKL) (He et al., 2009). Phosphorylated MLKL forms oligomers capable of acting as nonspecific pores in the cell membrane and cation channels, which disturb the normal cell dynamics and cause cell penetration and rupture (Adameova et al., 2017; Liao et al., 2017). Our present work reveals the potential for the treatment of OA by inhibiting necroptosis.

Selumetinib, a highly selective inhibitor of mitogen-activated protein kinase 1/2, is active against various tumors and has good therapeutic potential for several diseases. Selumetinib shows therapeutic effects on KRAS-mutated colorectal cancer (Yoon et al., 2011), non-small cell lung cancer (Imyanitov et al., 2020) metastatic melanoma (Patel and Kim, 2012). Recently, selumetinib has been approved for treating plexiform neurofibromas and has shown a variable treatment response (Gross et al., 2020; Errico et al., 2021). Selumetinib reduces the expression of angiotensin-converting enzyme 2 (ACE2) in human cells, which decreases the infectivity of SARS-CoV-2 (Zhou et al., 2020). In previous experiments using the Connectivity map database to screen for small molecules, selumetinib was found to exhibit expression opposite to RIP3 overexpression (Jeon et al., 2020). Selumetinib inhibits the MEK-ERK pathway and has neuroprotective effects on acrolein-induced neuroinflammation in central nervous system degenerative diseases (Ho et al., 2020). The potential mechanisms of the therapeutic effects of selumetinib in OA, however, remain unclear. Therefore, in our present study, we used IL-1β to stimulate ATDC5 cells to mimic the local inflammatory environment of cartilage *in vitro*. The receptor activator ligand of nuclear factor-κB (RANKL) was used to differentiate bone marrow macrophages (BMMs) into osteoclasts. We aimed to investigate the efficacy of selumetinib for treating OA and to provide new possibilities for its clinical application.

## Materials and methods

### Reagents and media

Selumetinib was purchased from Topscience Co., Ltd. (Shanghai, China). The fetal bovine serum (FBS) used was purchased from VWR (West Chester, PA, United States), the α-Minimum Essential Medium (α-MEM) and the Dulbecco’s modified Eagle’s medium (DMEM) was purchased from Biosharp (Anhui, China). IL-1β, TNF-α and Cell Counting Kit-8 (CCK-8) was obtained from MedChemExpress (New Jersey, United States). Nuclear factor-κB receptor activator ligand (RANKL) and mouse recombinant macrophage colony-stimulating factor (M-CSF) were purchased from LifeTein (Beijing, China). Collagen II monoclonal antibody (MA5-12789) and aggrecan neo polyclonal antibody (PA1-1746) were purchased from Thermo Fisher Scientific (Waltham, MA, United States). Anti-MMP9 (ab76003), anti-Adamts4 (ab185722), and anti-Adamts5 (ab185722) were purchased from Abcam (Cambridge, United Kingdom). Primary antibodies against ERK (4695), phosphorylated (p)-ERK (4370), JNK (9252S), P38 (9212S), P65 (8242), p-p65 (3033), iκBα (4812), p-iκBα (2859), RIP1 (3493), β-actin (4970) were purchased from Cell Signaling Technology (Danvers, MA, United States). SOX9 antibody (ET1611-56), p-P38 antibody (ER2001-52), p-JNK antibody (ET1601-28), RIP3 antibody (ET1902-67) were purchased from Huaan Biotechnology (Hangzhou, China). P-RIP1 antibody (AP1230), p-RIP3 antibody (AP1260), MLKL antibody (A19685), p-MLKL antibody (AP0949) were purchased from Abclonal (Wuhan, China).

### Molecular docking

Selumetinib molecular structures were obtained from the PubChem database (https://pubchem.ncbi.nlm.nih.gov/), formatted and energy minimised using Chem3D, and then imported into Schrodinger software to create the database. RIP3 (PDB ID: 6OKO) target protein structures were obtained from the RCSB database and processed using the Maestro 11.9 platform. Molecular docking was performed by the Glide module in the Schrödinger Maestro software. All molecules were prepared according to the default settings of the LigPrep module. Molecular docking and screening was performed by Standard Precision (SP) method to analyse the mode of interaction between the compounds and the target proteins, such as the resulting hydrogen bonding, π-π interactions, hydrophobic interactions, etc., and to speculate whether the compounds to be screened have certain active effects.

### Cell culture and treatment

6-week-old C57/BL6 mice were used to extract primary BMMs. After the mice were sacrificed, their bones were collected under aseptic conditions. The bone marrow was washed in a 100-mm petri dish with complete α-MEM containing 10% FBS, M-CSF(30 ng/mL) and 1% penicillin/streptomycin and incubated in an incubator with 5% CO_2_ for 4 days at 37°C or until 70% cell confluence was achieved. The culture medium was changed every alternate day to remove nonadherent cells. Adherent cells were considered as BMMs. The mouse chondrocyte ATDC5 cell line was obtained from the European Collection of Authenticated Cell Cultures. ATDC5 cells were cultured in DMEM containing 5% FBS and 1% penicillin/streptomycin in an incubator with 5% CO_2_ at 37°C. The medium was changed every alternate day until the cells achieved 80%–90% confluence. For IL-1β stimulation, the culture medium was replaced with DMEM containing IL-1β (10 ng/ml), and the cells were treated for 48 h. SW1353 cells were cultured in L-15 containing 10% FBS and 1% penicillin/streptomycin in an incubator with 5% CO_2_ at 37°C. Similarly, we treated SW1353 with 10 ng/ml IL-1β and different concentrations of selumetinib for 48 h.

### Cell viability assay

We assayed the effect of selumetinib on ATDC5 cells and BMMs by using the CCK-8 cell proliferation/cytotoxicity assay kit. ATDC5 cells were inoculated at the density of 1 × 10^3^ cells per well in 96-well plates containing complete DMEM, while BMMs were inoculated at a density of 6 × 10^3^ cells per well in complete α-MEM containing 30 ng/ml MCSF. After confirming cell adherence, the cells were treated with a serial dilution of selumetinib (from 2.5 to 80 nM) for 24, 48, 72, and 96 h. Following the completion of treatment of the cells, 10 μl of CCK-8 reagent was added to each well according to the instructions, and the cells were then placed in an incubator for 1 h. We then measured the absorbance at 450 nm by using a Multiskan FC microplate luminometer (Thermo Fisher Scientific, Waltham, MA, United States).

### High-density cell culture

We used a high-density culture to observe the ability of ATDC5 cells to secrete the ECM. A 10 μl suspension of ATDC5 cells with the density of 10^7^ cells/ml was inoculated at the bottom of a 24-well plate. After waiting for 6 h for the cells to fully adhere to the plate, we added DMEM complete medium containing 10% FBS and the corresponding drug (IL-1β 10 ng/ml; the concentration of selumetinib (1.25, 2.5, and 5 nM) was based on the previous CCK-8 experiment with ATDC5 cells); the medium was changed every alternate day, and the cells were incubated for 7 days. The cells were then fixed with 4% paraformaldehyde for 20 min. After 20 min, excess paraformaldehyde was washed off with phosphate-buffered saline (PBS) and the cells were stained with a toluidine blue staining kit for 5 min and then washed again. Finally, the 24-well plate was scanned and photographed using an Epson V600 Photo Scanner (Japan).

### *In vitro* osteoclast differentiation assay

To further investigate the effect of different concentrations of selumetinib on osteoclast differentiation *in vitro*, we inoculated M-CSF-dependent BMMs at a density of 6 × 10^3^ cells per well in 96-well plates. After microscopic confirmation of cell adherence, BMMs were treated with 50 ng/ml of RANKL and different concentrations of selumetinib (0, 10, 20, and 40 nM) simultaneously to observe the concentration-dependent effect of selumetinib. We also treated BMMs with 50 ng/ml of RANKL and 40 nM of selumetinib at different time periods of osteoblast differentiation (1–3 days, 3–5 days, and 5–7 days) to observe the time-dependent effect of selumetinib. All culture media were changed every alternate day until significant osteoclast formation was observed under the microscope in the control group. The cells were then fixed in 4% paraformaldehyde, and tartrate-resistant acid phosphatase (TRAP) staining was performed. The number of TRAP-positive osteoclasts containing three or more nuclei was counted, and the percentage of stained area per well was counted using ImageJ software (National Institutes of Health, Bethesda, MD).

### Quantitative real-time polymerase chain reaction analysis (qRT-PCR)

Chondrocytes were treated with IL-1β and different concentrations of selumetinib (0, 1.25, 2.5, and 5 nM) for 48 h according to the protocol of previous CCK-8 experiments. Bone marrow stromal cells were inoculated at the density of 3 × 10^5^ cells/well in 6-well plates. The cells were stimulated with 50 ng/ml RANKL and 30 ng/ml M-CSF along with various concentrations of selumetinib (0, 10, 20, and 40 nM) until osteoclasts were clearly formed in the control wells. Total RNA was extracted using TRIzol reagent (Gibco, United States). RNA was reverse transcribed to cDNA using the HiFiScript cDNA Synthesis kit (CWBiotech, China). The cDNA synthesis reaction conditions were incubated at 42°C for 15 min and 85°C for 5 min. ChamQ Universal SYBR qPCR Master Mix (Vazyme, China) and the ABI 7300 plus real-time PCR system (Applied Biosystems, Foster City, CA, United States) were used to perform qRT-PCR. The reaction conditions of qRT-PCR were as follows: pre-denaturation at 95°C for 30 s, cyclic reaction at 95°C for 10 s, 60°C for 30 s, repeated 40 times, melting curve 95°C for 15 s, 60°C for 60 s, and 95°C for 15 s. Relative expression levels were standardized to the expression of GAPDH mRNA and calculated using the 2^−ΔΔCT^ method. The primer sequences used were listed below: Aggrecan Forward 5′-AGG TGT CGC TCC CCA ACT AT-3′, Reverse 5′-CTT CAC AGC GGT AGA TCC CAG-3′, Collagen II Forward 5′-GGG TCA CAG AGG TTA CCC AG-3′, Reverse 5′- ACC AGG GGA ACC ACT CTC AC-3′, SOX9 Forward 5′-AGT ACC CGC ATC TGC ACA AC-3′, Reverse 5′- ACG AAG GGT CTC TTC TCG CT-3′, MMP9 Forward 5′-GGA CCC GAA GCG GAC ATT G-3′, Reverse 5′-CGT CGT CGA AAT GGG CAT CT-3′, ADAMTS4 Forward 5′-ATG GCC TCA ATC CAT CCC AG-3′, Reverse 5′- GCA AGC AGG GTT GGA ATC TTT G-3′, ADAMTS5 Forward 5′-CGC TAC ACT CTA AAG CCA CTC-3′, Reverse 5′- CCT CGA AGC TAA AGC CCT CG-3′, NFATc1 Forward 5′-CCG TTG CTT CCA GAA AAT AAC A-3′, Reverse 5′-TGT GGG ATG TGA ACT CGG AA-3′, cFos Forward 5′-CCA GTC AAG AGC ATC AGC AA-3′, Reverse 5′-AAG TAG TGC AGC CCG GAG TA-3′, ACP5 Forward 5′-CAC TCC CAC CCT GAG ATT TGT-3′, Reverse 5′-CCC CAG AGA CAT GAT GAA GTC A-3′, DC-STAMP Forward 5′-AAA ACC CTT GGG CTG TTC TT-3′, Reverse 5′-AAT​CAT​GGA​CGA​CTC​CTT​GG-3′, V-ATPase D2 Forward 5′-AAG CCT TTG TTT GAC GCT GT-3′, Reverse 5′-TTC GAT GCC TCT GTG AGA TG-3′, CTSK Forward 5′-CTT CCA ATA CGT GCA GCA GA-3′, Reverse 5′-TCT TCA GGG CTT TCT CGT TC-3′, V-ATPase D2 Forward 5′-AAG CCT TTG TTT GAC GCT GT-3′, Reverse 5′-TTC GAT GCC TCT GTG AGA TG-3′, GAPDH Forward 5′-ACC CAG AAG ACT GTG GAT GG-3′, Reverse 5′-ACC CAG AAG ACT GTG GAT GG-3’.

### Western blot analyses

To analyze the effect of selumetinib on RANKL-induced early BMM signaling pathways, BMMs inoculated in 6-well plates at a density of 3 × 10^5^ cells per well were pretreated for 1 h in the absence or presence of 40 nM selumetinib and then stimulated with 50 ng/ml RANKL for 5, 10, 20, 30, or 60 min. For RANKL-induced late signaling events, BMMs were cultured with intact α-MEM for 1, 3, or 5 days with or without 40 nM selumetinib. To investigate the dose-dependent effect of selumetinib on RANLK-related signaling events, BMM were incubated with intact α-MEM and selumetinib (0, 10, 20, or 40 nM) for 3 days to observe its effect on RANLK-related signaling events. To analyze the effect of selumetinib on the anabolic, catabolic, and related pathways in IL-1β-treated ATDC5 cells, the cells were simultaneously stimulated with different concentrations of selumetinib (0, 1.25, 2.5, and 5 nM) and IL-1β (10 ng/ml) for 48 h. For chondrocytes, the cells were stimulated with different concentrations of selumetinib (0, 1.25, 2.5, and 5 nM) and IL-1β (10 ng/ml) for 48 h, and the protein expression of anabolic, catabolic, and related pathways was measured by western blot.

After the culturing process, total protein of BMMs and ATDC5 was extracted with radioimmunoprecipitation assay (RIPA) buffer (Biosharp, Hefei, China) containing 1 mM phenylmethanesulfonyl fluoride (PMSF) and phosphatase inhibitors (MedChemExpress, NJ). The BCA protein assay kit (AMEKO, Shanghai, China) was used to determine the protein concentration. We separated 30 μg of protein by 10% SDS-PAGE gel electrophoresis (Epizyme, Shanghai, China) and transferred it to polyvinylidene fluoride (PVDF) membrane. The membrane was blocked with a blocking buffer (Biosharp) for 1–4 h at room temperature and incubated with the primary antibody in a refrigerator overnight at 4°C. The membrane was subsequently incubated with the secondary antibody for 1 h and then washed with Tris-buffered saline containing Tween (TBST). We used an ultrasensitive ECL chemiluminescence kit (NCM Biotech, Suzhou, China) and ImageQuant LAS 500 (GE Healthcare, Fairfield, CT, United States) to detect antibody reactivity. The intensity of each band was quantitatively analyzed by ImageJ software.

### Osteoarthritis model

To observe the protective effect of selumetinib on cartilage in animals, we chose the destabilization of the medial meniscus (DMM) approach to construct a knee osteoarthritis model. Ten-week-old male C57BL/6J mice were anesthetized, and a 3-mm longitudinal incision was made in the medial knee joint by using a scalpel. We then transected the medial meniscotibial ligament (MMTL) to reduce the stability of the knee joint. Finally, we sutured the inner joint capsule and closed the skin. The sham procedure involved opening the joint capsule and then closing the incision without cutting the MMTL. Twenty-four mice were randomly divided into four groups of six mice each. Group 1 mice were subjected to sham surgery and received PBS on alternate days. The right knee joint of mice in groups 2–4 was subjected to DMM surgery. After surgery, different drugs were administered intraperitoneally for 4 weeks. Group 2 mice received PBS on alternate days, group 3 received selumetinib at a dose of 2 mg/kg body weight, and group 4 received selumetinib at a dose of 10 mg/kg body weight.

### Histological observations

We fixed the knee joints of each group in 4% paraformaldehyde for 24 h and decalcified them with 10% EDTA solution for 4 weeks. Serial sections (4 μm thickness) were taken from the anterior part of the joint and stained with hematoxylin and eosin (H&E) and Senna O-fast green (S-O) to visualize cartilage destruction. The extent of cartilage degeneration was assessed based on the Osteoarthritis Research Society International (OARSI) scoring system. TRAP staining was performed to visualize the effect of selumetinib on osteoclast formation *in vivo*. Three sections from each group were taken for quantitative analysis, and the images were analyzed by ImageJ.

### Statistical analysis

**FIGURE 1:**
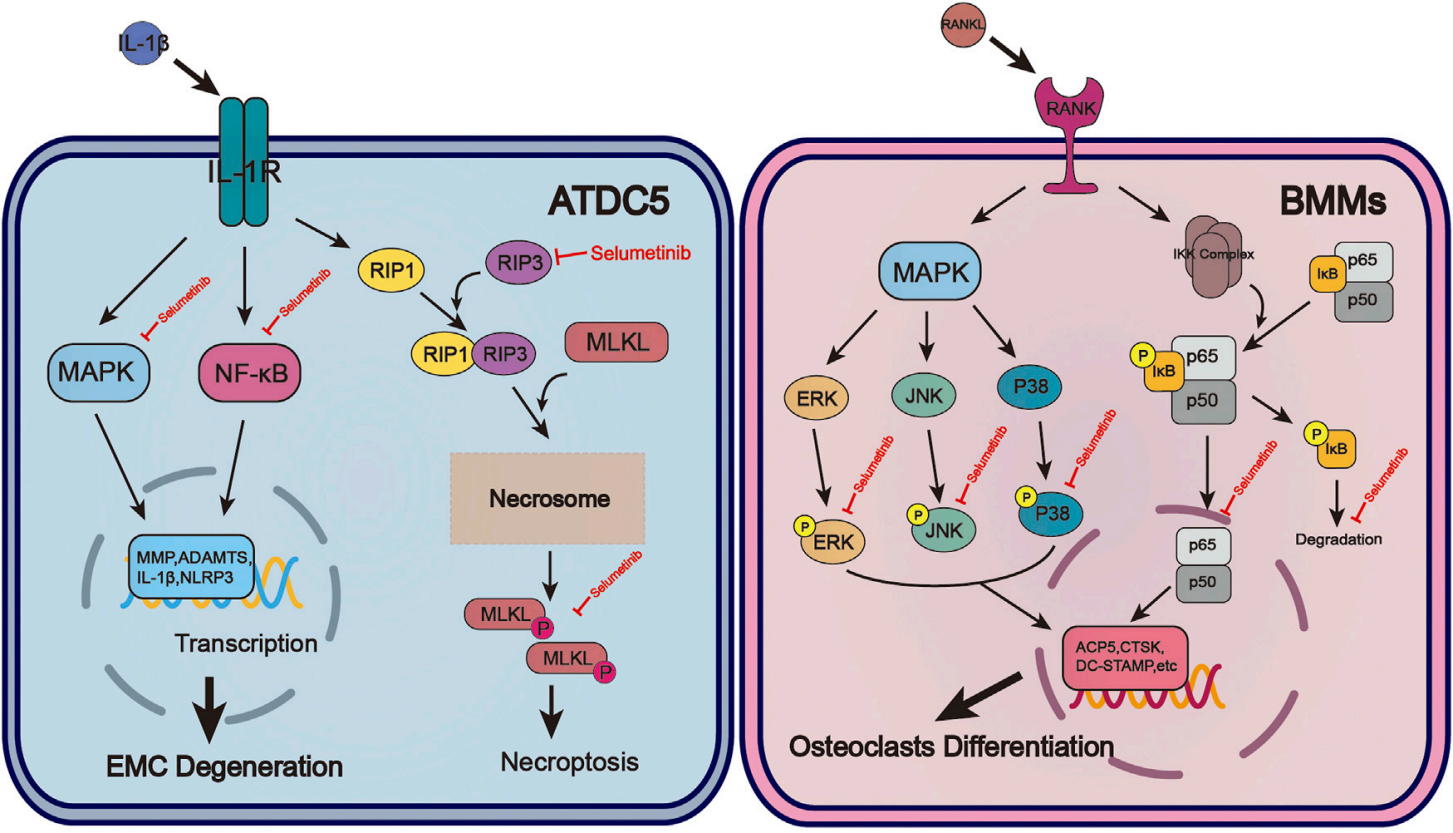
The molecular mechanism of selumetinib protects chondrocytes and inhibits osteoclast formation during OA. In ATDC5, selumetinib inhibited IL-1β-induced activation of NF-kB and MAPK signalling pathways and alleviated EMC degeneration. Selumetinib also attenuated the occurrence of inflammation by inhibiting necroptosis. For BMMs, selumetinib inhibited the activation of NF-kB and MAPK signaling pathways induced by RANKL, reducd osteoclast-associated gene expression and osteoclast formation.

Three repetitions of the experiment were performed for each group. The experimental data were expressed as mean ± standard deviation. An independent sample Student’s t-test was used to compare the data between the two groups. One-way analysis of variance was used for comparisons between multiple groups. Statistical analysis of the data was performed using GraphPad Prism 5.0 (GraphPad Software, La Jolla, CA, United States). A P value of < 0.05 was considered to be statistically significant (Figure 1) (Pereira et al., 2011; Zhang et al., 2015b).

## Results

### Molecular docking analysis between selumetinib and RIP3

**FIGURE 2:**
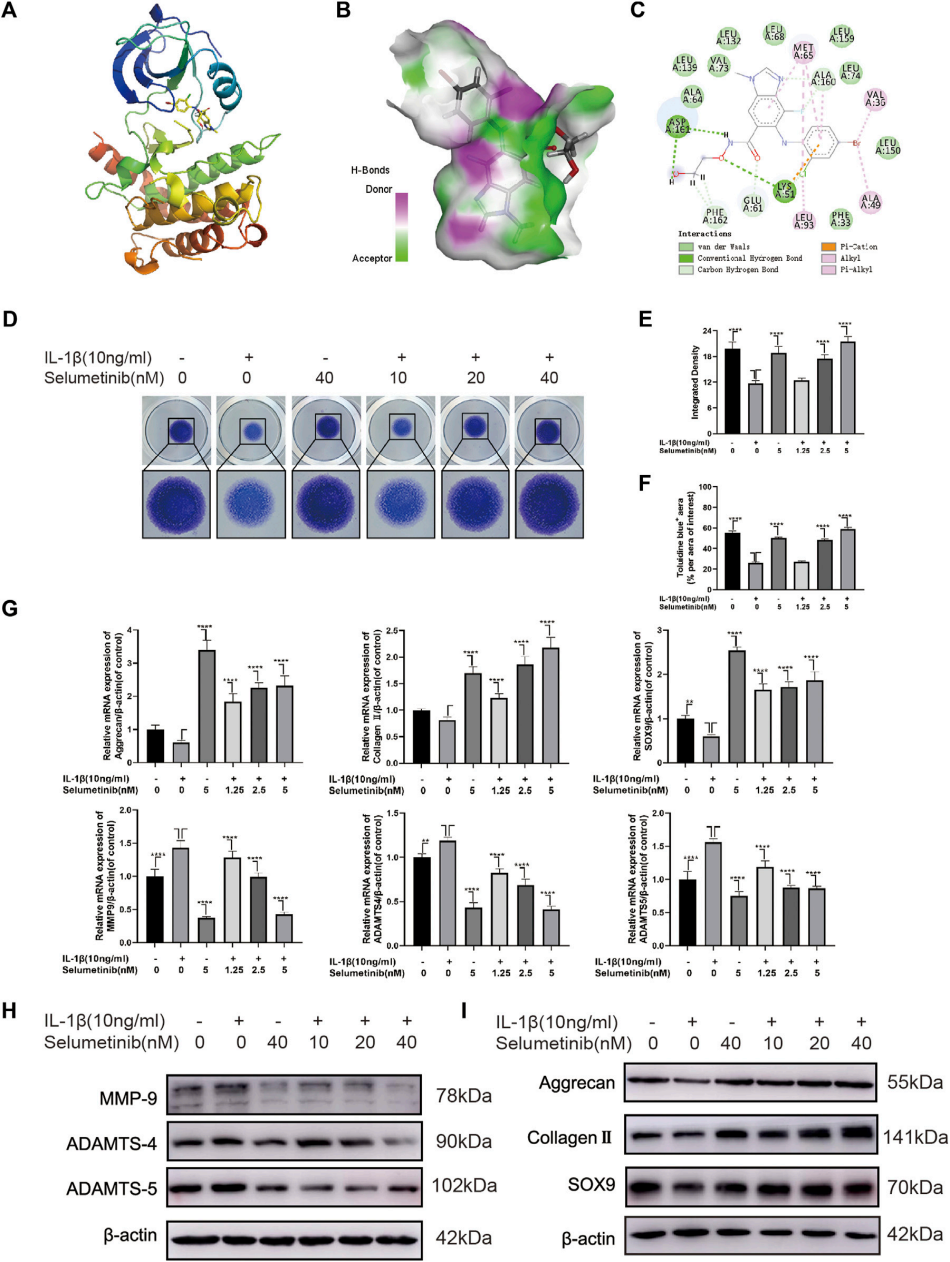
Selumenitib inhibits ECM degradation and promotes ECM synthesis in IL-1β-treated ATDC5 cells. **(A–C)** We performed a molecular docking between Selumetinib and RIP3 and displayed the detail binding mode. **(D)** We performed Toluidine blue staining on high-density cultured ATDC5 cells after treatment with different concentrations of Selumetinib (0, 1.25, 2.5, or 5 nM) and IL-1β (10 ng/ml) for 7 days. **(E,F)** The integrated density value and Toluidine blue (+) aera (% per aera of interest) were performed to evaluate the extracellular matrix (ECM) of ATDC5 cells after the indicated treatment. We stimulated ATDC5 cells with different concentrations of Selumetinib (0, 1.25, 2.5 or 5 nM) and IL-1β (10 ng/ml) for 48 h to observe the effects of Selumetinib on ECM synthesis and catabolism. **(G–I)** The expression levels of anabolic-related genes (Collagen II, Aggrecan and SOX9) and catabolism-related genes (MMP9, ADAMTS-4 and ADAMTS-5) were evaluated by quantitative real-time polymerase chain reaction (RT-qPCR) and Western Blot. Data are presented as mean ± S.D. n = 3. Significant differences between groups are indicated as ****p < 0.0001, ***p < 0.001, **p < 0.01, *p < 0.05.

Previous experiments by Jimin Jeon et al. (2020) using the Connectivity map database to screen for small molecules, selumetinib was revealed to exhibit an opposite expression to RIP3 overexpression. Hence, we performed a molecular docking analysis of the compound selumetinib with the RIP3 target protein to verify the correlation between this two molecules. The chemical structure of selumetinib is shown in Supplementary Figure S1A. The results showed that Selumetinib was able to bind well to the RIP3 target protein with a good match (binding energy of −8.51 kcal/mol, less than −5 kcal/mol). The docked complexes were visualised using Pymol 2.1 software to obtain the binding pattern of the compound to the protein (Figure 2A). As shown in Figures 2B,C, the amino acid residues in which selumetinib interacts with the active site of the RIP3 protein are VAL-73, ALA-64, MET-65, ALA-160, VAL-36, ALA-49, LEU-93, ASP-161, LYS-51 and others. Selumetinib contains two benzene rings and a five-membered carbon heterocycle, which are highly hydrophobic. Its benzene ring is able to form strong conjugation interactions with active site amino acids (VAL-73, ALA-64, MET-65, ALA-160, VAL-36, ALA-49, LEU-93), which are important for stabilizing small molecules in the protein cavity. In addition, selumetinib is able to form two hydrogen bonding interactions with amino acids ASP-161 and LYS-51, which contribute to the anchoring of small molecules in the protein cavity. In summary, selumetinib has shown good performance in docking and scoring and binding modes with RIP3 target proteins, forming stable complexes with proteins that are strongly associated with RIP3 targets.

### Effects of selumetinib on IL-1β-induced ECM synthesis and degradation

First, we used the CCK-8 cell viability/cytotoxicity assay to determine the safe concentration range of selumetinib for ATDC5 cells. Cells were cultured in selumetinib at increasing concentrations (0, 2.5, 5, 10, 20, 40, and 80 nM) for 24 h. Supplementary Figure S1B shows that cell viability was not significantly affected at selumetinib concentrations up to 80 nM. When the cells were treated for 48 h, selumetinib at 10 nM and higher concentrations significantly inhibited cell proliferation, while 5 nM and lower concentrations of selumetinib showed no significant cytotoxic effect. Thus, the concentrations of selumetinib used in the subsequent experiments were 0, 1.25, 2.5, and 5 nM.

To study the effects of selumetinib on ECM synthesis and degradation, we used a high-density culture of ATDC5 cells and toluidine blue staining (Figure 2D). The integrated density and area of ECM of ATDC5 cells were significantly reduced following IL-1β treatment. However, the loss of ECM was reversed after selumetinib treatment (Figures 2E,F). Simultaneously, we treated ATDC5 cells with different concentrations of selumetinib and IL-1β for 48 h, followed by western blot analysis to detect the protein expression of the synthesis and catabolism-related factors. As shown in Figure 2H and Supplementary Figure S1C, IL-1β-induced expression of catabolic-related proteins (MMP9, ADAMTS-4, and ADAMTS-5) was partially inhibited by selumetinib treatment. Regarding anabolism, selumetinib promoted the expression of type II collagen, aggrecan, and SOX9 (Figure 2I, Supplementary Figure S1D), which was consistent with qRT-PCR results (Figure 2G). In order see whether selumetinib has the same protective effect on human-derived chondrocytes, we treated SW1353 cells with IL-1β and examined the expression of anabolic and catabolic-related proteins (Supplementary Figures S1H,I). The results showed that selumetinib can alleviate ECM degradation and promote synthesis in both ATDC5 and SW1353 cells.

### Effects of selumetinib on necroptosis-related signaling pathways ATDC5 cells

**FIGURE 3:**
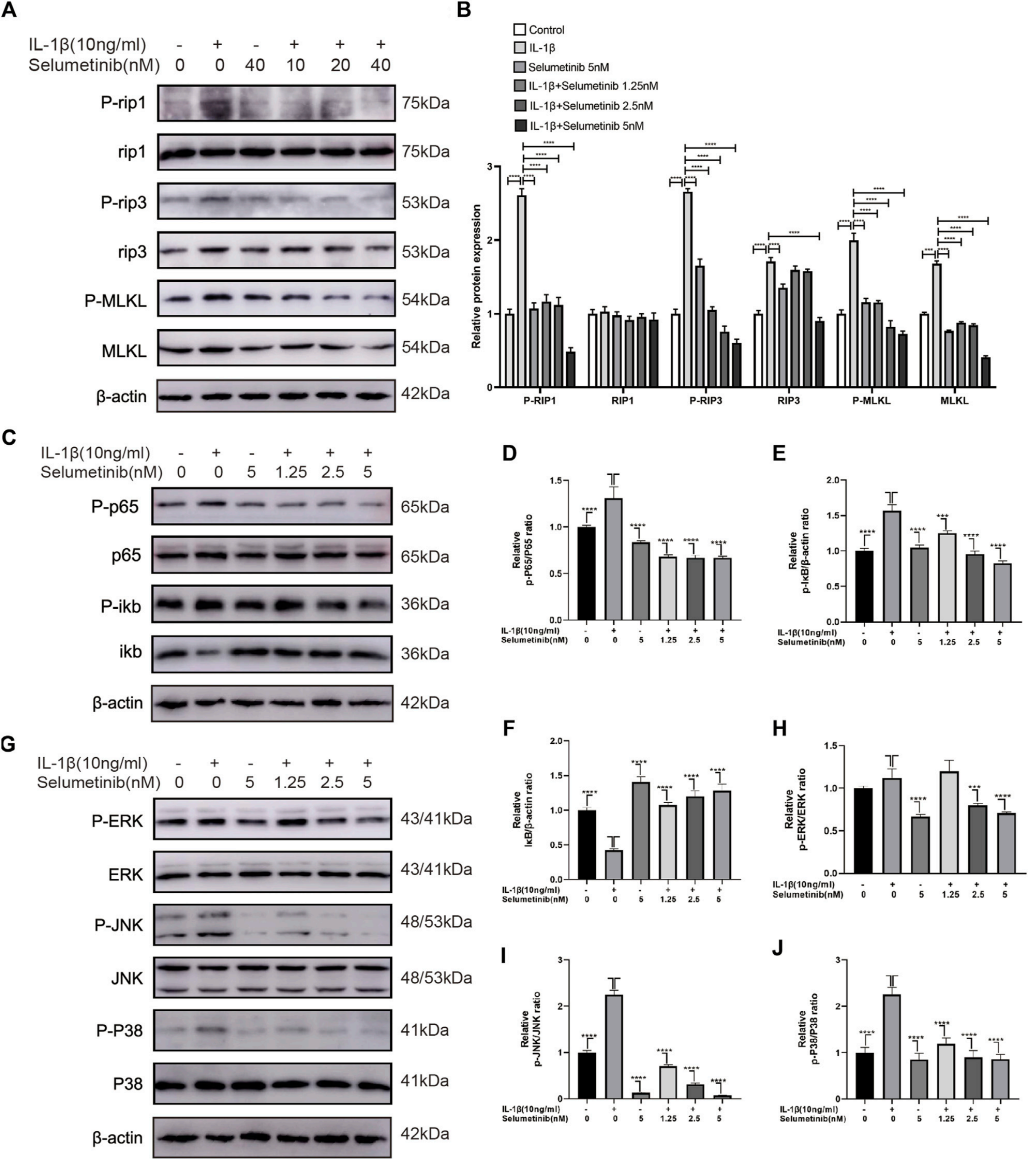
Selumetinib inhibits IL-1β-induced activation of necroptosis-related, MAPK and NF-κB signalling pathways and attenuates inflammatory responses. **(A,B)** Western blot revealed the effect of Selumteinib on necroptosis-related proteins, including p-RIP1/RIP1, p-RIP3/RIP3, p-MLKL/MLKL. **(C–F)** Selumetinib affected the NF-κB pathway-related proteins, including p-IκBα, IκBα, p-p65 and p65. **(G–J)** The effects of selumetinib on MAPK signalling pathway proteins, including p-ERK/ERK, p-JNK/JNK and p-p38/p38. Data are presented as mean ± S.D. n = 3. Significant differences between groups are indicated as ****p < 0.0001, ***p < 0.001, **p < 0.01, *p < 0.05.

Necroptosis, a specialized pathway of cell death, is involved in the occurrence of OA, which can interfere with normal chondrocyte viability and induce structural and functional defects of cartilage (Cheng et al., 2021). Figures 3A,B shows the expression levels of necroptosis-related proteins such as phospho-RIP1, RIP1, phospho-RIP3, RIP3, phospho-MLKL, and MLKL. In the IL-1β group, the expression levels of p-RIP1, RIP3, p-RIP3, MLKL, and p-MLKL were increased. Under selumetinib treatment, these necroptosis-related proteins were inhibited, particularly at the concentrations of 2.5 and 5 nM. In addition, we used TNF-α (10 ng/ml) as a stimulus to observe the effect of different concentrations of selumetinib on necroptosis-related signaling pathways. The results were consistent with previous experiments (Supplementary Figure S2A,B). Taken together, these results indicate that selumetinib alleviates the occurrence of inflammation and delays the degradation of chondrocyte matrix by inhibiting RIP1/RIP3/MLKL signaling pathways in ATDC5 cells.

### Effects of selumetinib on the NF-κB and MAPK signaling pathways in ATDC5 cells

Previous studies have reported that the NF-κB pathway is associated with IL-1β-stimulated inflammatory response (Kim et al., 2018; Lu et al., 2021). To further investigate whether selumetinib exerts protective effects through the NF-κB pathway, we detected the activation and phosphorylation of this pathway by western blot assay. Figures 3C–F shows that the protein expression of p-p65 and p-IκB was significantly higher in the IL-1β-treated group than in the untreated group. However, selumetinib treatment significantly reduced the expression of p-p65 and p-IκB, and increased the expression of IκB.

In the development of osteoarthritis, the MAPK pathway has been demonstrated to have a critical role in modulating ECM metabolism and inflammation. (Maudens et al., 2018; Zhou et al., 2019). We further investigated the protective effect of selumetinib in the activation of the MAPK signaling pathway induced by IL-1β. The data in Figure 3G show that IL-1β rapidly activated the MAPK signaling pathway and increased the expression levels of phosphorylated ERK, JNK, and p38. Selumetinib inhibited this phenomenon in a dose-dependent manner. Statistical analysis of western blot assay results is presented in Figures 3H–J. In short, selumetinib suppresses inflammatory responses by inhibiting the NF-κB and MAPK signaling pathways.

### Selumetinib inhibits RANKL-induced osteoclast formation and osteoclast-related gene expression *in vitro*

We treated BMMs with different concentrations of selumetinib for 24, 48, 72, or 96 h and determined cell viability by the CCK-8 assay. The results are presented in Supplementary Figure S2C and show that selumetinib at 40 nM and lower levels had no toxic effects on the cells at the four time points. However, selumetinib at 80 nM concentration exerted an inhibitory effect on the cell growth of BMMs. Therefore, in the subsequent experiments, we chose selumetinib concentrations of 10, 20, and 40 nM for treating BMMs.

**FIGURE 4:**
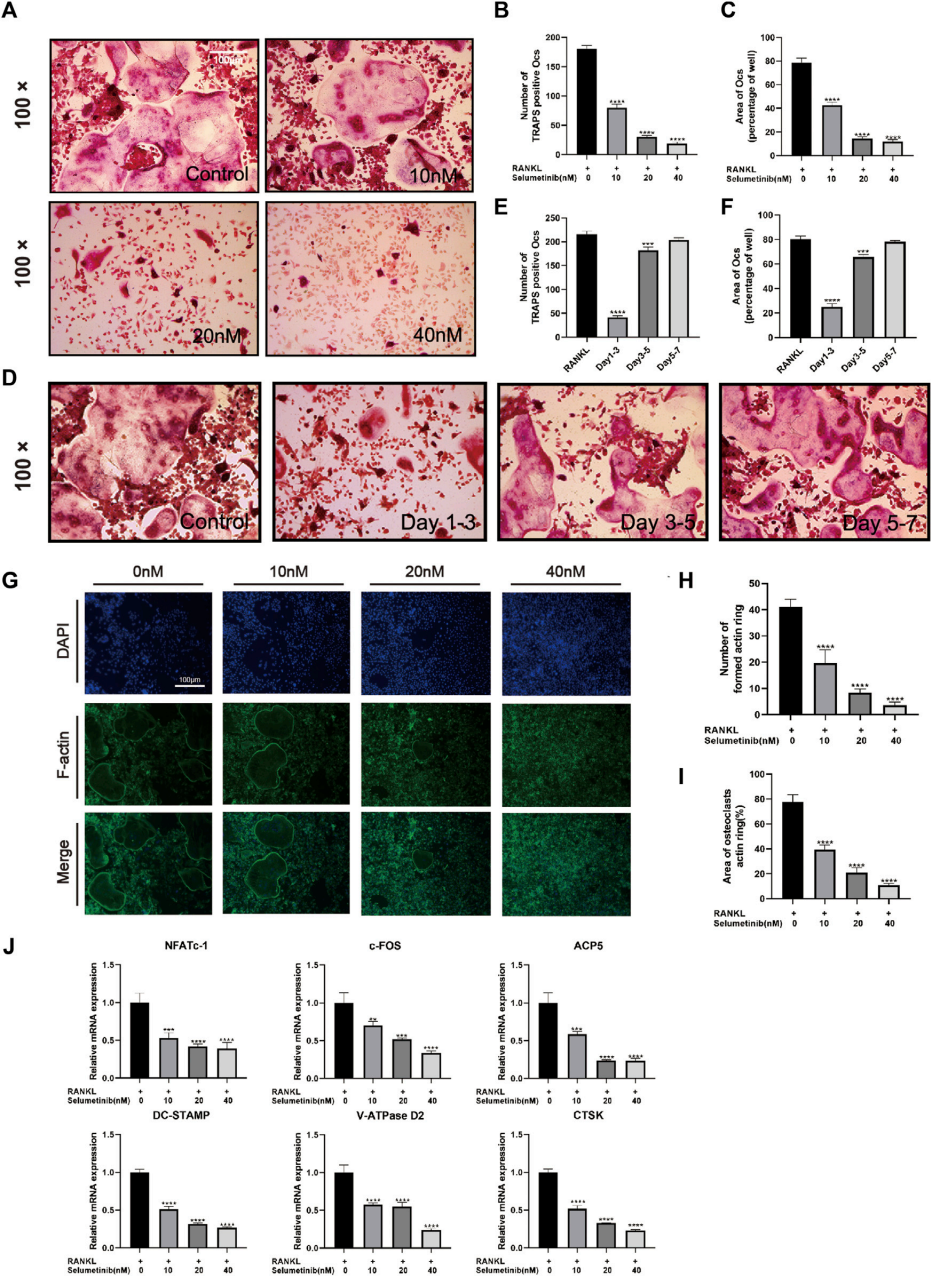
Selumetinib inhibits RANKL-induced osteoclast-related gene expression and osteoblast differentiation *in vitro*. **(A)** We stimulated BMMs with MCSF (30 ng/mL), RANK (50 ng/mL) and different concentrations of selumetinib (10, 20, and 40 nM) for 7 days to investigate the concentration-dependent inhibition of osteoblast differentiation by selumetinib. **(B,C)** The area and number of TRAP-stained positive cells (nuclei ≥ 3) were recorded. **(D–F)** The time-dependent inhibition of osteoclast formation was investigated by treating 40 nM selumetinib at different time points (days 1-3, 3-5 or 5-7) to counteract the effect of RANKL on promoting osteoclast differentiation. **(G)** We stained BMMs for laser scanning confocal microscopy observation and quantified the number of formed actin rings and the area of osteoclasts actin ring (%) **(H,I)**. **(J)** We treated BMMs with RANKL and 10, 20 and 40 nM selumetinib for 5 days and performed RT-PCR to examine osteoclast-related mRNA expression levels, including NFATc1, c-fos, ACP5, DC-STAMP, V-ATPase D2 and CTSK. Data are presented as mean ± S.D. n = 3. Significant differences between groups are indicated as ****p < 0.0001, ***p < 0.001, **p < 0.01, *p < 0.05.

To investigate whether selumetinib has a significant effect on osteoclast differentiation, we treated BMMs with both RANKL and selumetinib for 7 days. In this experiment, selumetinib concentrations were guided by the previous CCK-8 experiments. When there were numerous huge osteoclasts generated in the RANKL-treated group, we fixed the cells with 4% paraformaldehyde and performed TRAP staining. To investigate whether the effect of selumetinib on osteoclasts is dependent on concentration, we treated BMMs with different concentrations of selumetinib. The number of osteoclasts that stained positive for TRAP and the mean area occupied by osteoclasts decreased under treatment with different concentrations of selumetinib and demonstrated a dose-dependent therapeutic effect (Figures 4A–C). In addition, we stained BMMs for laser scanning confocal microscopy observation. The number and size of F-actin belt also showed a dose-dependent decline under selumetinib treatment (Figures 4G–I). Selumetinib was added at different periods of RANKL stimulation to observe its time-dependent effect on osteoclast differentiation. Treatment with 40 nM of selumetinib on days 1–3 significantly inhibited osteoclastogenesis, but the same dose of selumetinib did not produce a significant protective effect in the middle or late stages (Figures 4D–F).

The following genes for osteoclast differentiation and fusion were detected by RT-PCR: c-Fos, nuclear factor of activated T cells, cytoplasmic 1 (NFATC1), tartrate-resistant acid phosphatase 5 (ACP5), Vacuolar-type H-ATPase D2(V-ATPase D2), dendritic cell-specific transmembrane protein (DC-STAMP), and cathepsin k (CTSK). Consistent with previous experiments, selumetinib inhibited the expression of osteoclast-associated genes in a dose-dependent manner (Figure 4J). In summary, selumetinib could significantly inhibit osteoclast formation and reduce osteoclast marker genes expression *in vitro*.

### Selumetinib suppresses osteoclastogenesis by inhibiting the NF-κB and MAPK signaling pathways

**FIGURE 5:**
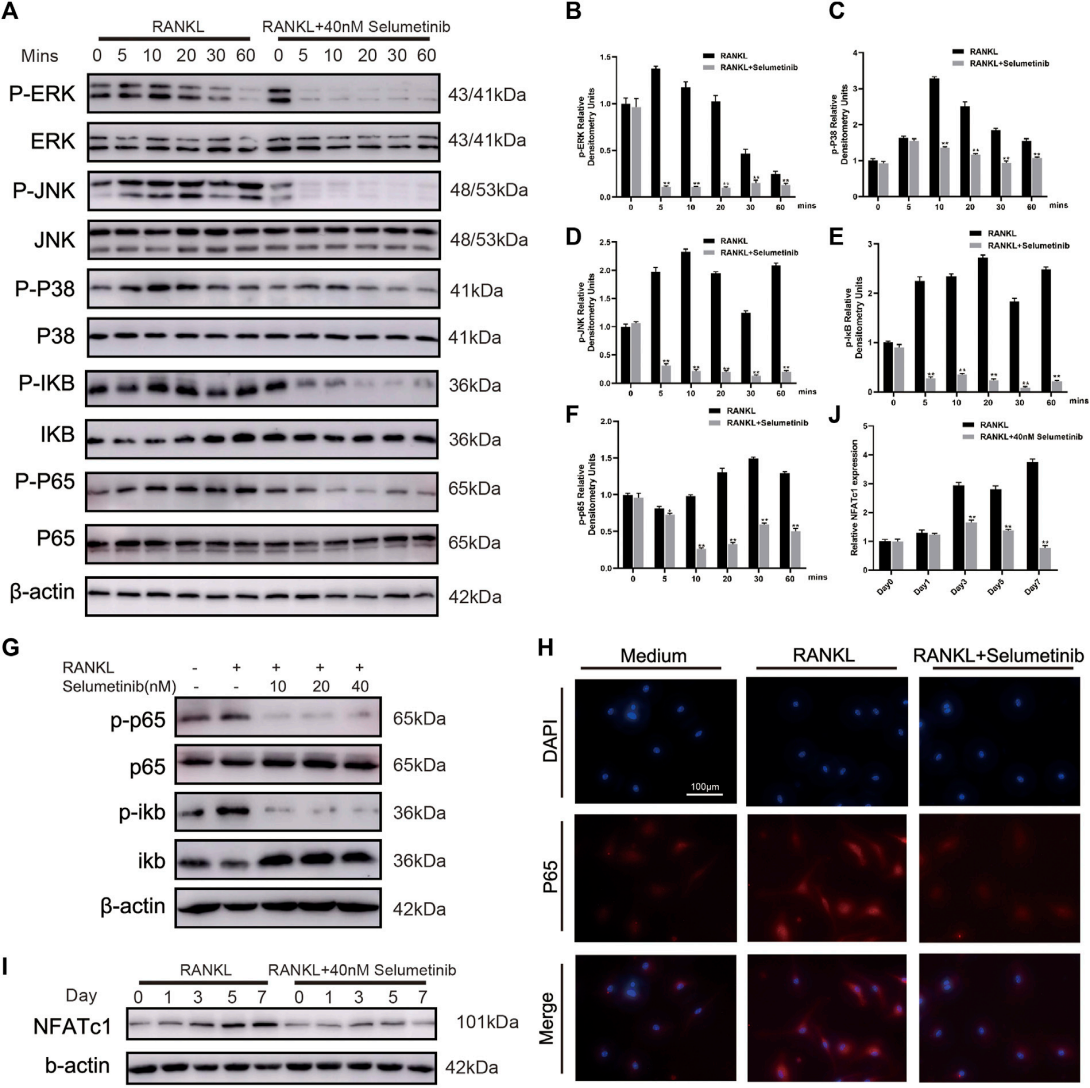
Selumetinib impairs RANKL-induced activation of the MAPK and NF-κB signalling pathway. **(A)** BMMs were pretreated for 1 h with or without selumetinib (40 nM) and then stimulated by RANKL (50 ng/mL) for 0, 5, 10, 20, 30 or 60 min. The proteins expression of NF-kB and MAPK signalling pathway was assessed by Western blot analysis, and quantified by image J **(B–F)**. **(G)** BMMs were treated with RANKL (50 ng/mL) and different concentrations of selumetinib (10, 20 or 40 nM) simultaneously for 3 days to observe the effect of different concentrations of selumetinib on the NF-kB pathway. **(H)** After treatment with RANKL (50 ng/ml) without or with selumetinib (40 nM), BMMs were stained for laser scanning confocal microscopy assay. **(I,J)** Treatment with 50 ng/mL RANKL and 40 nM Selumetinib simultaneously for 1, 3 or 5 days to observe the relative expression of NFATc1. Data are presented as mean ± S.D. n = 3. Significant differences between groups are indicated as ****p < 0.0001, ***p < 0.001, **p < 0.01, *p < 0.05.

**FIGURE 6:**
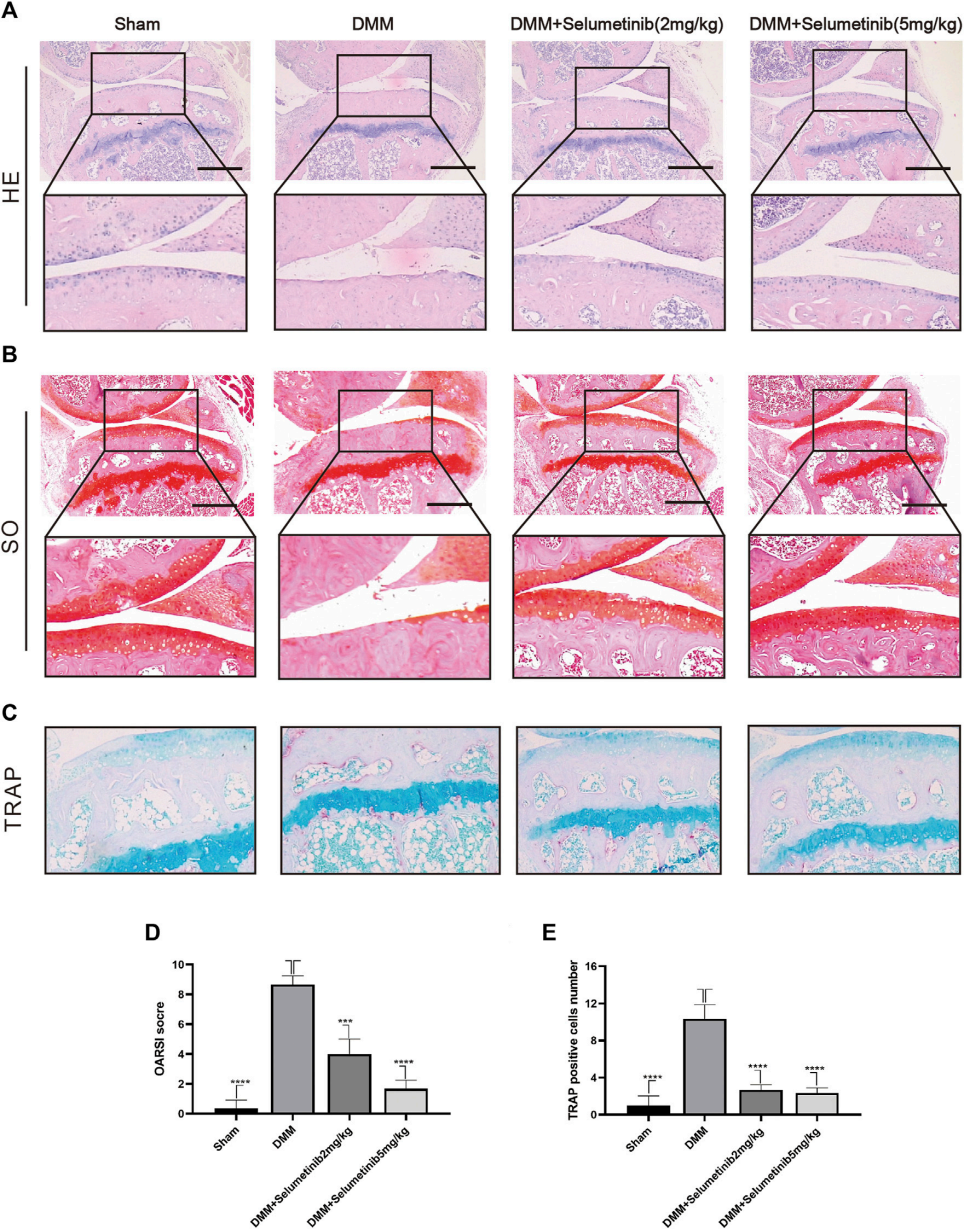
Selumenitib suppresses the degradation of cartilage and osteoclasts induced by DMM *in vivo*. To observe the protective effect of different concentrations of Selumetinib on OA, we stained sections of mouse knee joints with Safranin O staining **(A)** and hematoxylin and eosin (H&E) staining **(B)** to observe the cartilage and performed OARSI scores **(D)** to compare the histological differences between different experimental groups. **(C)** TRAP staining was used to observe osteoclast formation in subchondral bone and the number of TRAP positive cells was recorded **(E)**. Data are presented as mean ± S.D. n = 3. Significant differences between groups are indicated as ****p < 0.0001, ***p < 0.001, **p < 0.01, *p < 0.05.

The NF-κB and MAPK signaling pathways have been proved to be involved in osteoclast differentiation (Jiang et al., 2020; Liao et al., 2021). As shown in Figure 5A, a short stimulation of RANKL could induce the activation of IκBα phosphorylation, leading to its degradation. At the same time, the phosphorylation expression of P65 increased. After selumetinib treatment, the NF-κB signaling pathway was significantly inhibited, and phosphorylation of IκBα and p65 was reduced. Regarding the dose-dependent effect of selumetinib, the NF-κB pathway showed activation after 3 days of RANKL stimulation, while the higher concentration of selumetinib had a stronger inhibitory effect (Figure 5G, Supplementary Figure S2D). We used confocal microscopy to observe the nuclear translocation of P65. The results showed that nuclear translocation of P65 caused by RANKL stimulation was significantly inhibited by selumetinib (Figure 6H).

The stimulation of RANKL simultaneously affects the MAPK signaling pathway (Figure 5A). Phosphorylation of ERK, JNK, and P38 was increased after short-term RANK treatment. This effect was attenuated by selumetinib, wherein the activation of all three MAPKs was inhibited. NFATC1 is closely associated with osteoclast production (Chen et al., 2017; Cao et al., 2019), and the activation of the NF-κB and MAPK signaling pathways is essential for NFATC1 expression. Western blot results showed a significant increase in NFATC1 expression after RANKL stimulation for 5 or 7 days and a significant decrease after treatment with 40 nM selumetinib (Figures 5I,J). This finding was consistent with previous RT-PCR results (Figure 4J). Therefore, we conclude that selumetinib treatment inhibits the activation of NF-κB and MAPK, resulting in a decrease in the expression of NFATC1.

### Selumetinib suppresses the degradation of cartilage and osteoclasts induced by DMM *in vivo*

We used DMM surgery to establish a mouse model of OA to investigate whether selumetinib has a protective effect against OA progression *in vivo*. We selected two concentrations of selumetinib for intraperitoneal injection: 2 mg/kg and 10 mg/kg. Selumetinib was injected every alternate day for 4 weeks. As shown in Figure 6A, H&E staining in the DMM group showed significant cartilage destruction and reduced cartilage thickness. S-O staining also showed loss of proteoglycans and a higher OARSI score in the DMM group (Figures 6B,D). Selumetinib treatment at both high and low concentrations showed a significant protective effect. The cartilage surface was relatively smoother, and the damaged articular cartilage was repaired.

**FIGURE 7:**
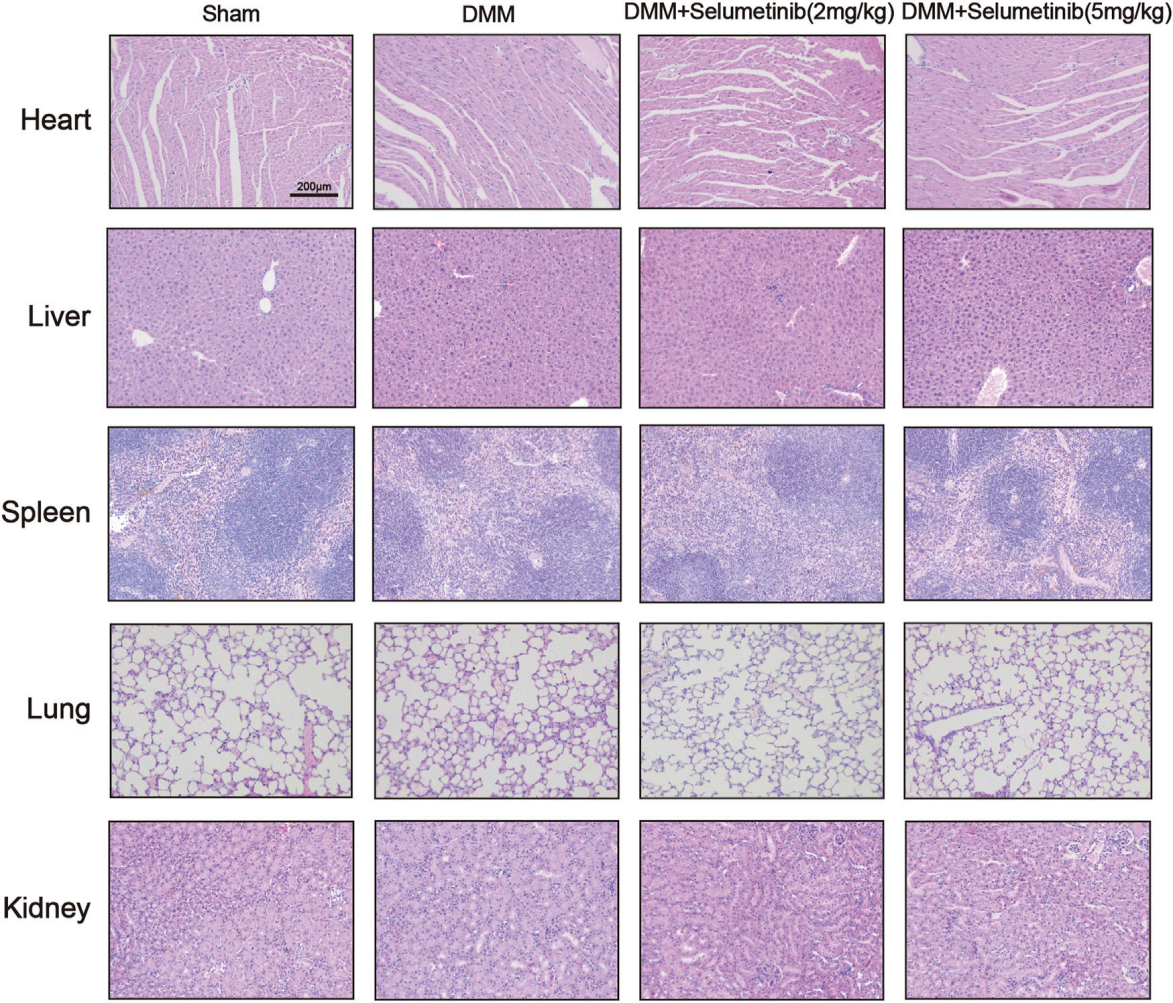
HE staining showed that Selumetinib had no obvious toxic effects on the heart, liver, spleen, lung, kidney *in vivo*.

We performed TRAP staining on slices to observe the protective effect of selumetinib on subchondral bone. The DMM group showed a significant increase in the number of TRAP-positive mature osteoclasts. In contrast, the number of mature osteoclasts decreased under selumetinib treatment (Figures 6C,E). In addition, to verify the toxic effects of selumetinib *in vivo*, we performed HE staining on the heart, liver, spleen, lungs and kidneys of the mice in the treatment group (Figure 7). The results showed that selumetinib had no significant organotoxic effects in both the low and high concentration groups.

Taken together, our results demonstrate that selumetinib can alleviate the progression of OA by inhibiting cartilage destruction and osteoclast formation. Thus, selumetinib has potential as a therapeutic agent for OA.

## Discussion

OA is a widespread and disabling disease characterized by cartilage degeneration, subchondral osteosclerosis, and synovitis (A et al., 2011, Ahedi et al., 2020). The necroptosis of chondrocytes and the degradation of the ECM together contribute to the development of osteoarthritis (Cheng et al., 2021; Li et al., 2021). Traditional drugs only alleviate clinical symptoms temporarily, but cannot efficiently treat OA (Zeng et al., 2018). More effective agents are therefore urgently required to attenuate the development of OA. Selumetinib has shown activity and positive therapeutic potential against a wide range of tumors. (Kang et al., 2018; Pellegrini et al., 2019; Peng et al., 2020; Wu et al., 2020). Therefore, we investigated the potential mechanisms underlying the protective effect of selumetinib on the development of OA.

High expression levels of inflammatory cytokines (including IL-1 and TNF-α) have been reported to play an important role in the development of cartilage destruction (Guan et al., 2018; Wang and He, 2018). IL-1β, a major inflammatory and catabolic cytokine in the pathophysiology of osteoarthritis, has been widely used to research. Several studies have revealed that necroptosis signaling induces the activation of the RIPK3-MLKL-NLRP3-Caspase-1 axis, leading to the conversion of pro-IL-1β to mature IL-1β, ultimately causing an enhanced inflammatory response and increased tissue damage (2015, Conos et al., 2017). We investigated whether selumetinib exerts a counteracting effect on IL-1β *in vitro* and delays the development of DMM-induced OA. Not surprisingly, after treatment with IL-1β, the expression levels of necroptosis-related proteins (P-MLKL, P-RIP1, and R-RIP3) were significantly increased. These changes were partly reversed by selumetinib. The qRT-PCR results agreed with these findings. In conclusion, selumetinib can inhibit the necroptosis of chondrocytes and delay cartilage destruction.

The ability of chondrocytes to synthesize and secrete ECM (mainly composed of aggrecan and type II collagen) isimportant for their normal physiological function (Li et al., 2019; Chen et al., 2021). Sox-9 is one of the key regulators of chondrocyte-specific matrix components, which can actively participate in the regulation of type II collagen and aggrecan (Zhang et al., 2015a; Yang et al., 2017b). Enzymatic proteins such as MMP-9, ADAMTS-4, and ADAMTS-5 are also involved in the maintenance of normal cartilage function, and these proteins are involved in decomposing the components of the cartilage matrix (Sellam and Berenbaum, 2010; Yang et al., 2017a; Chang et al., 2018). There is considerable evidence that the expression of MMPs and ADAMTS increases under IL-1β stimulation (Ismail et al., 2015; Beltrami-Moreira et al., 2016). Our data indicate that selumetinib inhibited the production of MMP9, ADAMTS-4, and ADAMTS-5 and alleviated the degradation of type II collagen and aggrecan in osteoarthritic chondrocytes, thereby maintaining the equilibrium between the disruption and reconstruction of the ECM.

Mechanistically, the NF-κB pathway is a classical pathway in OA development, which modulates inflammation and promotes osteoclast formation, chondrocyte apoptosis, and ECM degradation (Wu et al., 2019; Qiu et al., 2020; Shao et al., 2020; Liao et al., 2021). In cells stimulated by RANKL, the IκB protein is phosphorylated by IκB kinase (IKK) and degraded through the proteasome pathway (Liu et al., 2021). This leads to the phosphorylation of P65, which translocates into the nucleus and attaches to certain DNA sites to initiate the transcription of osteoclast-related genes (Zhu et al., 2016; Ilchovska and Barrow, 2020). NF-κB is also involved in the regulation of inflammatory signals and up-regulates the expression of IL-1β and TNF-α (Barnes and Karin, 1997; Khan and Khan, 2018; Hunto et al., 2020). In our present study, selumetinib inhibited IκBα degradation and attenuated the activation of p65 phosphorylation, Ultimately, this process reduces the transcriptional activity of pro-inflammatory cytokines (Khan and Khan, 2018).

MAPKs, including c-Jun NH2-terminal kinase (JNK), p38 MAPK, and extracellular signal-regulated kinase (ERK), regulate various cellular activities and maintain normal physiological functions (Kim and Choi, 2015; Liao et al., 2021). The compromised MAPK signaling pathways have been reported to contribute to the pathological process in various diseasesincluding cancer (Wei et al., 2021), autoimmune diseases (Banerjee et al., 2021), coronary heart diseases (Yang et al., 2018) and neurodegenerative disorders (Zhan-qiang et al., 2021). Inhibitors of MAPK have the potential to prevent osteoporosis and subchondral bone destruction during OA (Jie et al., 2018; Wang et al., 2019; Zhao et al., 2019). The MAPK and NF-κB signaling pathways are associated with the expression of osteoclastogenic transcription factors such as c-Fos and NFATc1. Among these, NFATc1 is a master transcription factor for the downstream effector of RANKL and can lead to increased expression of various osteoclast-related genes (Cao et al., 2019; Wang et al., 2020). We found that selumetinib treatment could inhibit RANKL-induced activation of NFATc1 and reduce osteoclast formation.

In our present study, we demonstrated that selumetinib has shown good performance in docking and scoring and binding modes with RIP3 target proteins, forming stable complexes with proteins that are strongly associated with RIP3 targets. Selumetinib reduces cartilage inflammation caused by IL-1β treatment and maintains ECM homeostasis by inhibiting the necroptosis in ATDC5 cells. It also reduces RANKL-induced differentiation of BMMs to osteoclasts and inhibits the transcription and expression of osteoclast-related genes. We also found that selumetinib exerts these protective effects by inhibiting the MAPK and NF-κB signaling pathways. This finding suggests that selumetinib has the potential to be a promising drug for treating OA. The present study has some limitations. The mechanism of action of selumetinib needs to be further investigated. The present study focused on the effects of selumetinib on MAPK and NF-κB, but it is unclear whether other pathways are also involved. The cells used in our experiments were mainly derived from mice and human; the clinical value of selumetinib remains to be further investigated.

## Data Availability

The datasets presented in this study can be found in online repositories. The names of the repository/repositories and accession number(s) can be found in the article/Supplementary Material.
